# Supplementary material for: Rational design of a multi-valent human papillomavirus vaccine by capsomere-hybrid co-assembly of virus-like particles
Source: Nat Commun. 2020 Jun 5;11:2841. doi: 10.1038/s41467-020-16639-1 (PMC7275066; doi:10.1038/s41467-020-16639-1)
Supplement: Supplementary file 1 — Supplementary Information [file 41467_2020_16639_MOESM1_ESM.pdf]

Supplementary information

Rational design of a multi-valent human papillomavirus vaccine by capsomere-hybrid co-assembly of virus-like particles

Wang et al.

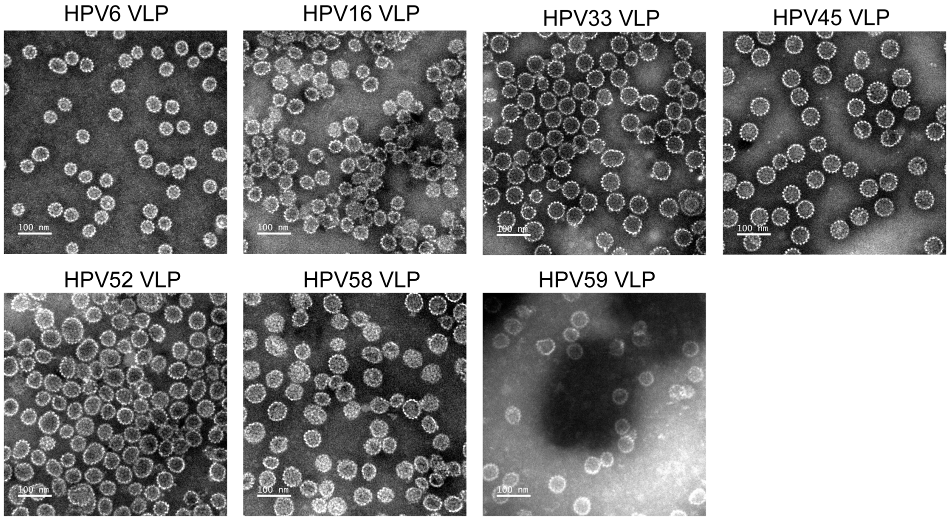

Supplementary Figure 1 Transmission electron microscopy (TEM) images of the purified HPV6, -16, -33, -45, -52, -58, and -59 L1 VLPs used in this study. Scale bar, 100 nm. One representative image from three biological repeats is shown.

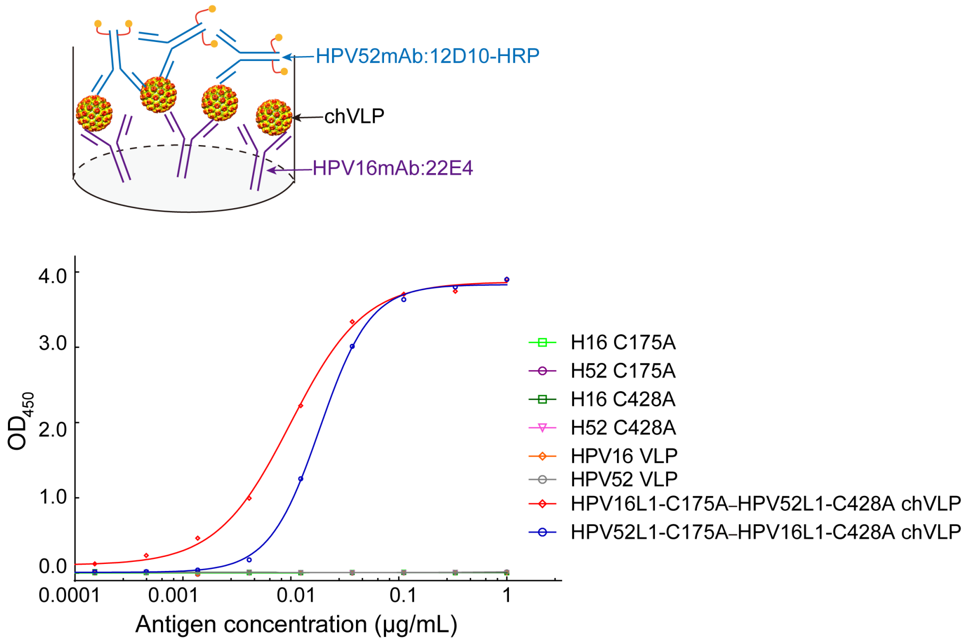

Supplementary Figure 2 Antigenicities of HPV16L1-C175A–HPV52L1-C428A chVLPs and HPV52L1-C175A–HPV16L1-C428A chVLPs were determined using sandwich ELISA. HPV C175A or C428A mutants and WT VLPs served as the control. Data were obtained from single experiment. The schematic diagram (top) shows the sandwich ELISA with 22E4 as the capturing antibody and 12D10-HRP as the detection antibody.

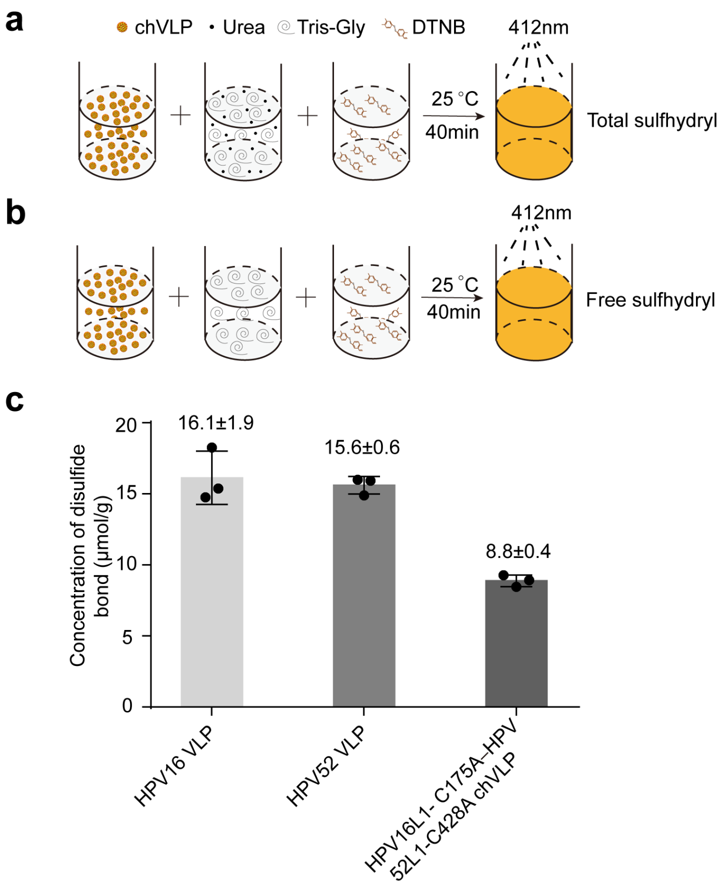

Supplementary Figure 3 Disulfide bonding measurement of HPV16L1-C175A–HPV52L1-C428A chVLPs and WT VLPs. Schematic diagram of the detection of the total sulfhydryl (SH) content (a) and free SH content (b) of HPV VLPs. (c) The concentration of disulfide bonds for HPV16L1-C175A–HPV52L1-C428A chVLPs, WT HPV16, and HPV52 VLPs. Data are presented as mean ± SD (n = 3). Source data are provided as a source data file.

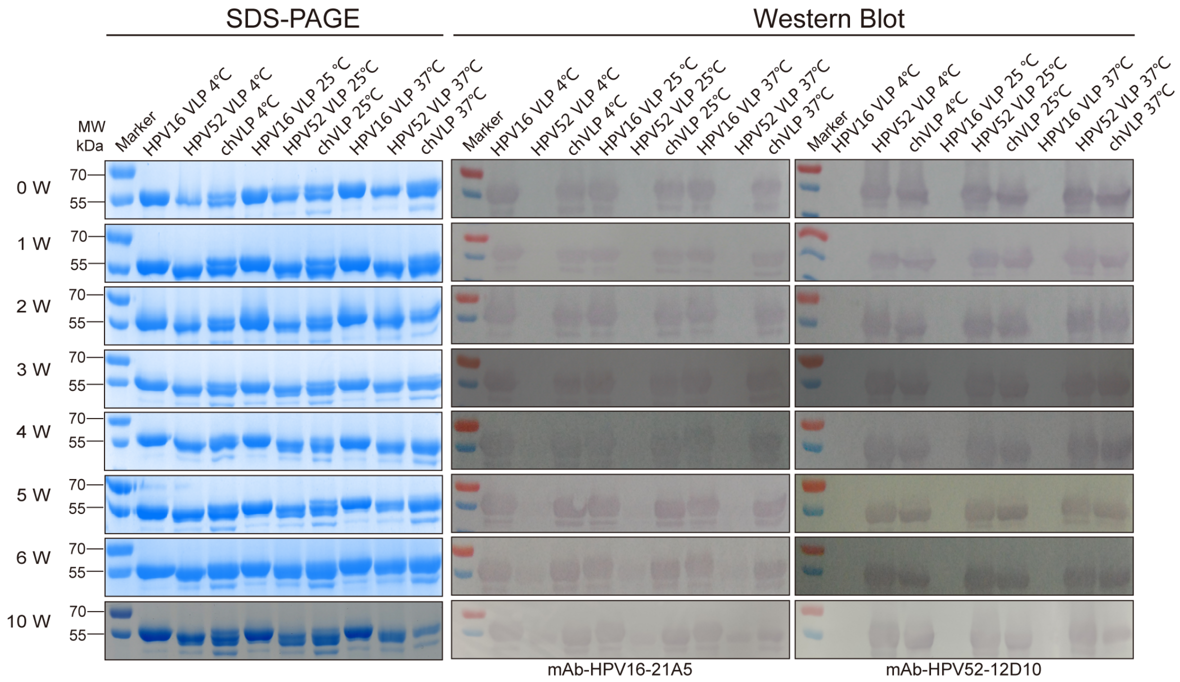

Supplementary Figure 4 Protein integrity assessment to characterize the stability of HPV16, -52 VLPs and HPV16L1-C175A–HPV52L1-C428A chVLPs. HPV16, -52, and HPV16L1-C175A–HPV52L1-C428A chVLPs were incubated at three temperatures for 10 weeks and subjected to reducing SDS-PAGE and western blotting with HPV16- or HPV52-L1-specific mAbs (21A5 or 12D10). All samples showed unchanged protein integrity across the long-term incubation. The uncropped original scans can be found in Supplementary Fig. 18.

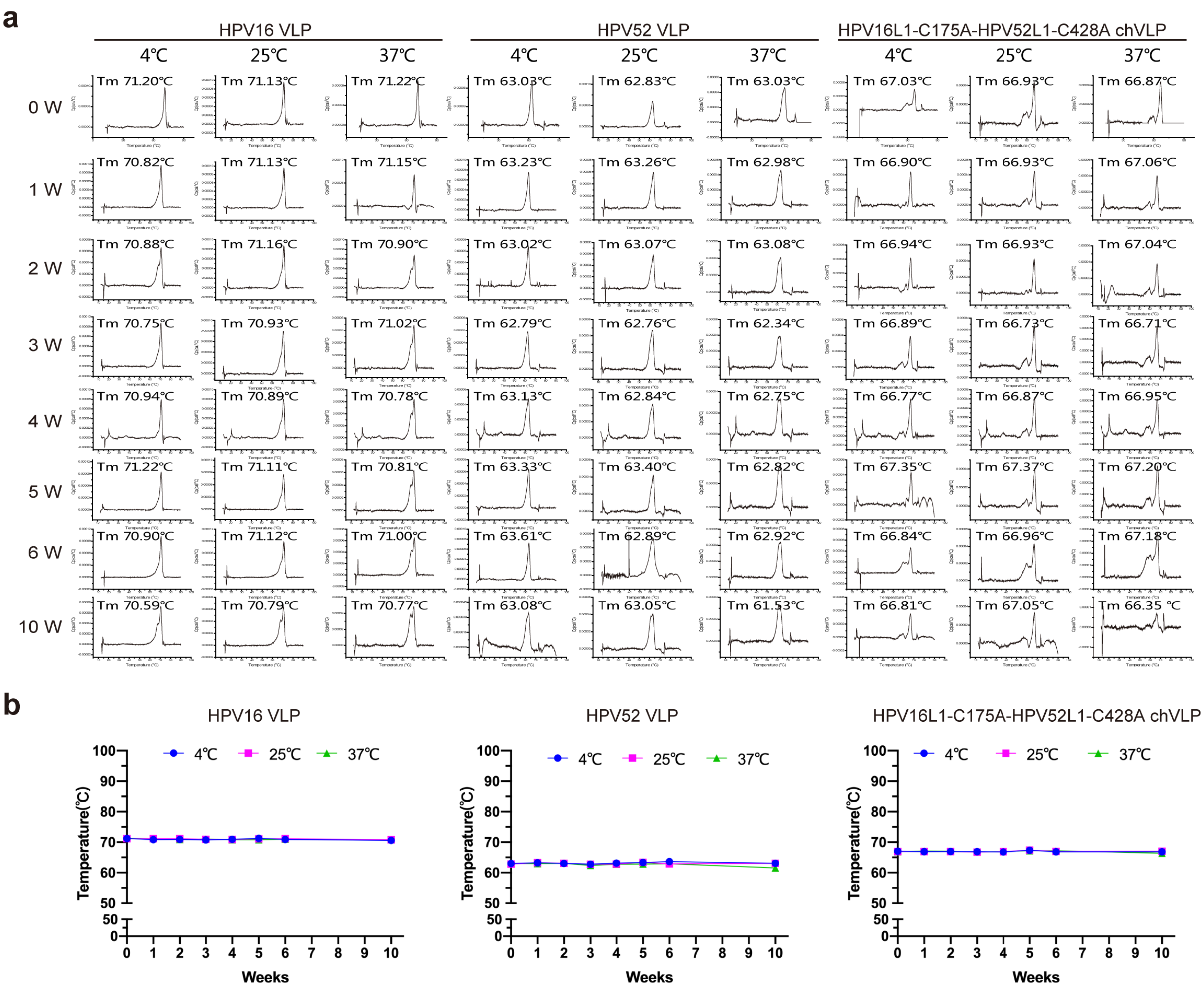

Supplementary Figure 5 Differential scanning calorimetry (DSC) analysis to characterize the stability of HPV16, -52 VLPs and HPV16L1-C175A–HPV52L1-C428A chVLPs. (a) HPV16, -52 VLPs and HPV16L1-C175A–HPV52L1-C428A chVLPs appeared stable under different thermal conditions. Single thermal transition peaks in the DSC profiles are shown, with unchanged Tm values of approximately 71°C, 63°C, and 67°C, respectively. (b) Tm values were plotted against incubation time. The fitted curves show excellent stability for chVLPs as well as the WT VLPs. Data were obtained from single experiment.

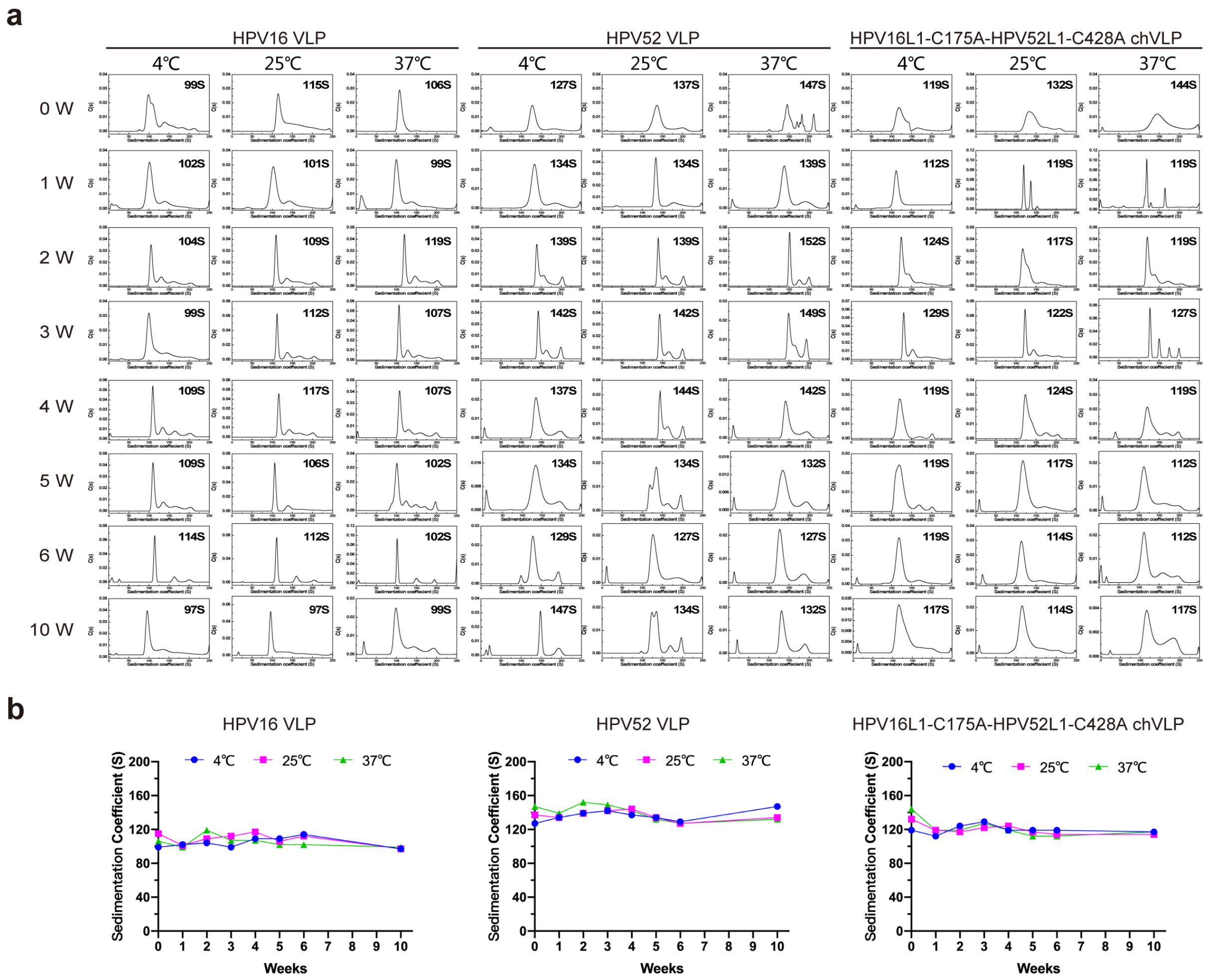

Supplementary Figure 6 Analytical ultra-centrifugation (AUC) sedimentation analysis to characterize the stability of HPV16, -52 VLPs and HPV16L1-C175A–HPV52L1-C428A chVLPs. (a) C(s) profiles measured by sedimentation velocity method in AUC. (b) The sedimentation coefficient was plotted against incubation time. The results show minor variations in the sedimentation coefficients for the chVLPs and WT VLPs over the 10-week incubation period at 4°C, 25°C, and 37°C. Data were obtained from single experiment.

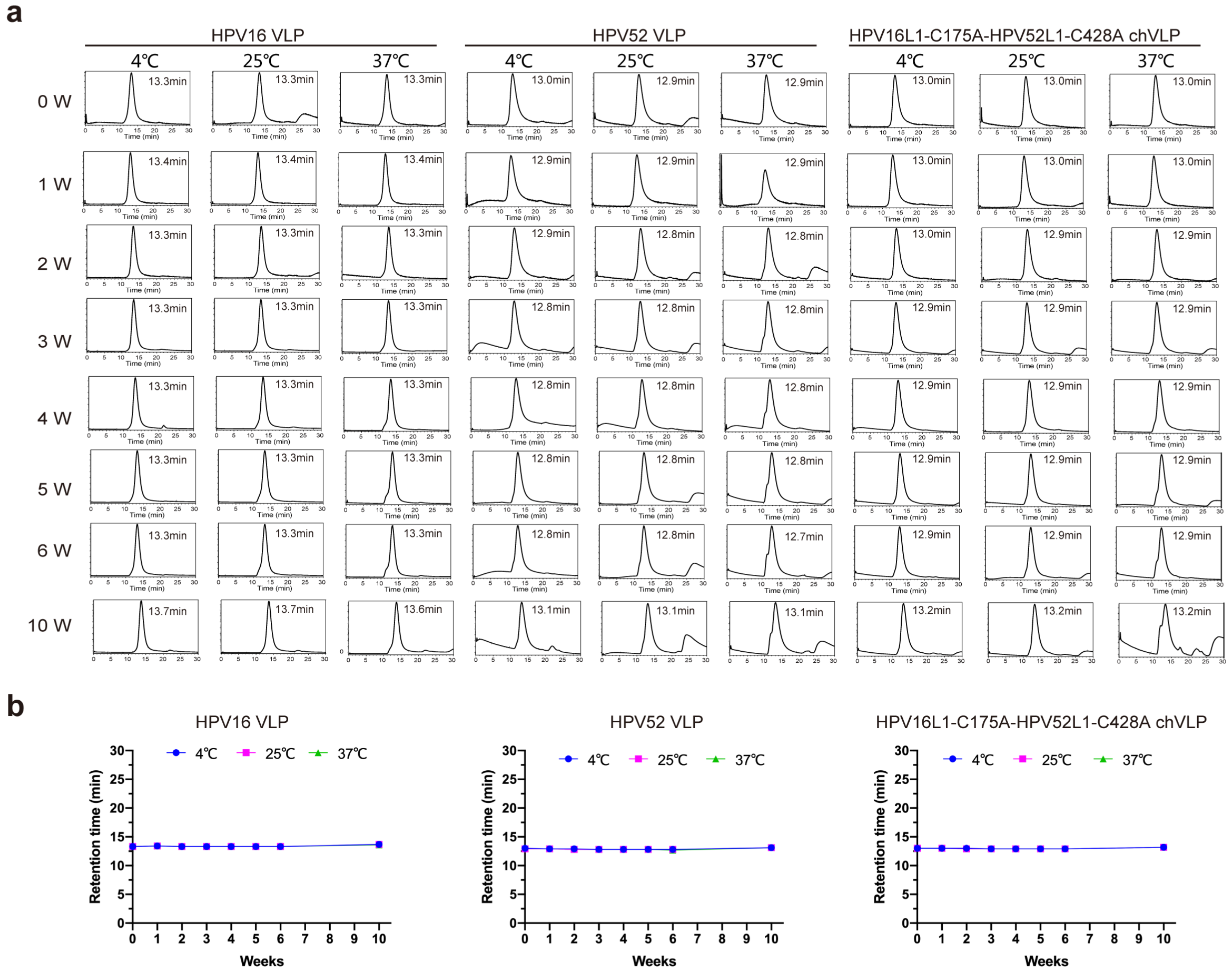

Supplementary Figure 7 High-performance size-exclusion chromatography (HPSEC) analysis to characterize the stability of HPV16, -52 VLPs and HPV16L1-C175A – HPV52L1-C428A chVLPs. (a) The HPSEC profiles of the samples incubated for 10 weeks at 4°C, 25°C and 37°C. (b) The retention times were plotted against incubation time. HPV16L1-C175A–HPV52L1-C428A chVLP and WT VLPs exhibited unchanged particle properties. Data were obtained from single experiment.

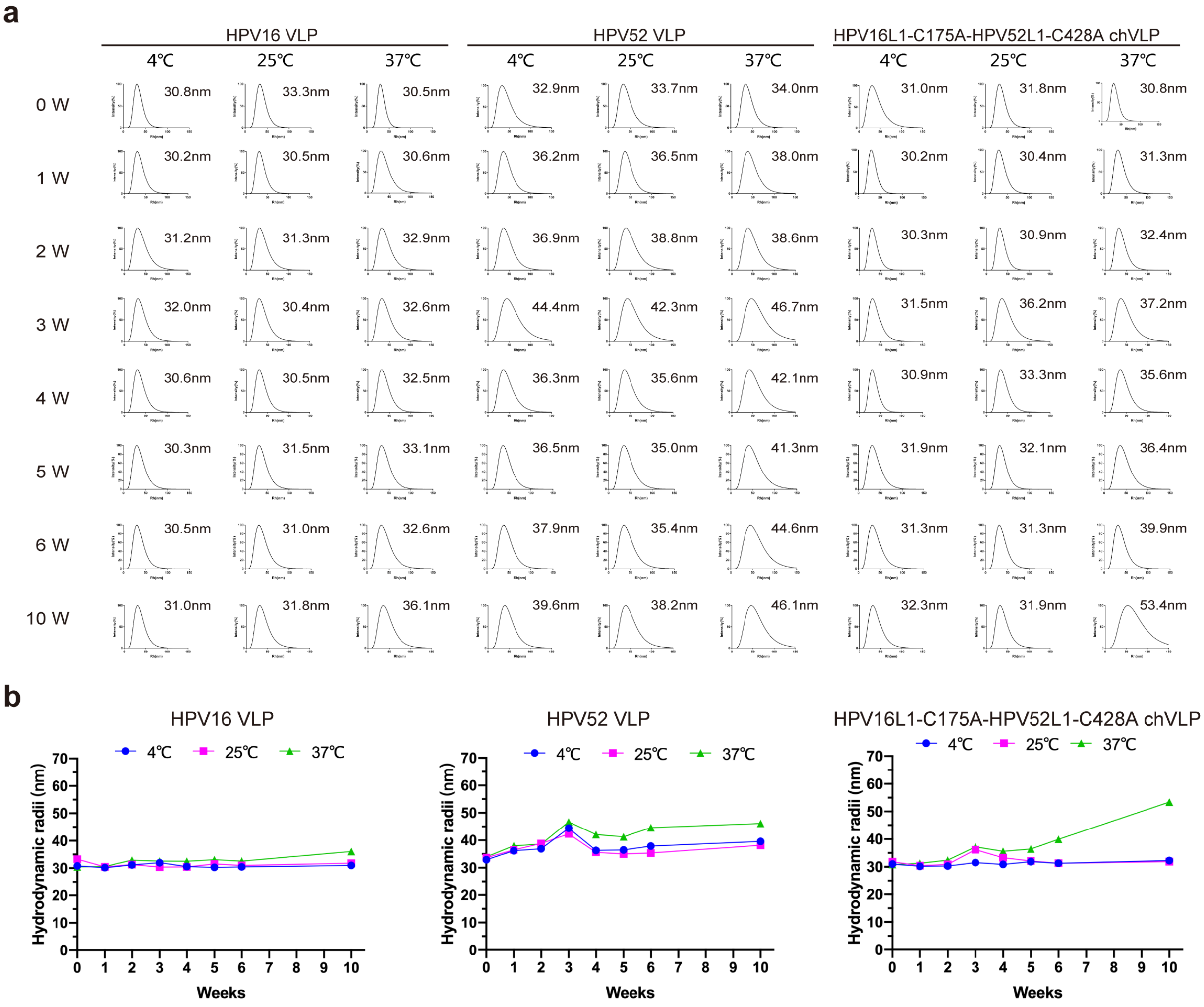

Supplementary Figure 8 Dynamic light scattering (DLS) analysis to characterize the stability of HPV16, -52 VLPs and HPV16L1-C175A–HPV52L1-C428A chVLPs. (a) DLS was used to measure the hydrodynamic radii (nm) of the main components of the VLP samples. (b) Hydrodynamic radii plotted against incubation time. HPV16, -52 VLPs and HPV16L1-C175A–HPV52L1-C428A chVLPs maintained minor particle size vibrations for 6 weeks, with the hydrodynamic radii increasing at week 10, particularly for chVLPs. This suggests that chVLPs have good stability for at least the first 6 weeks of storage. Data were obtained from single experiment.

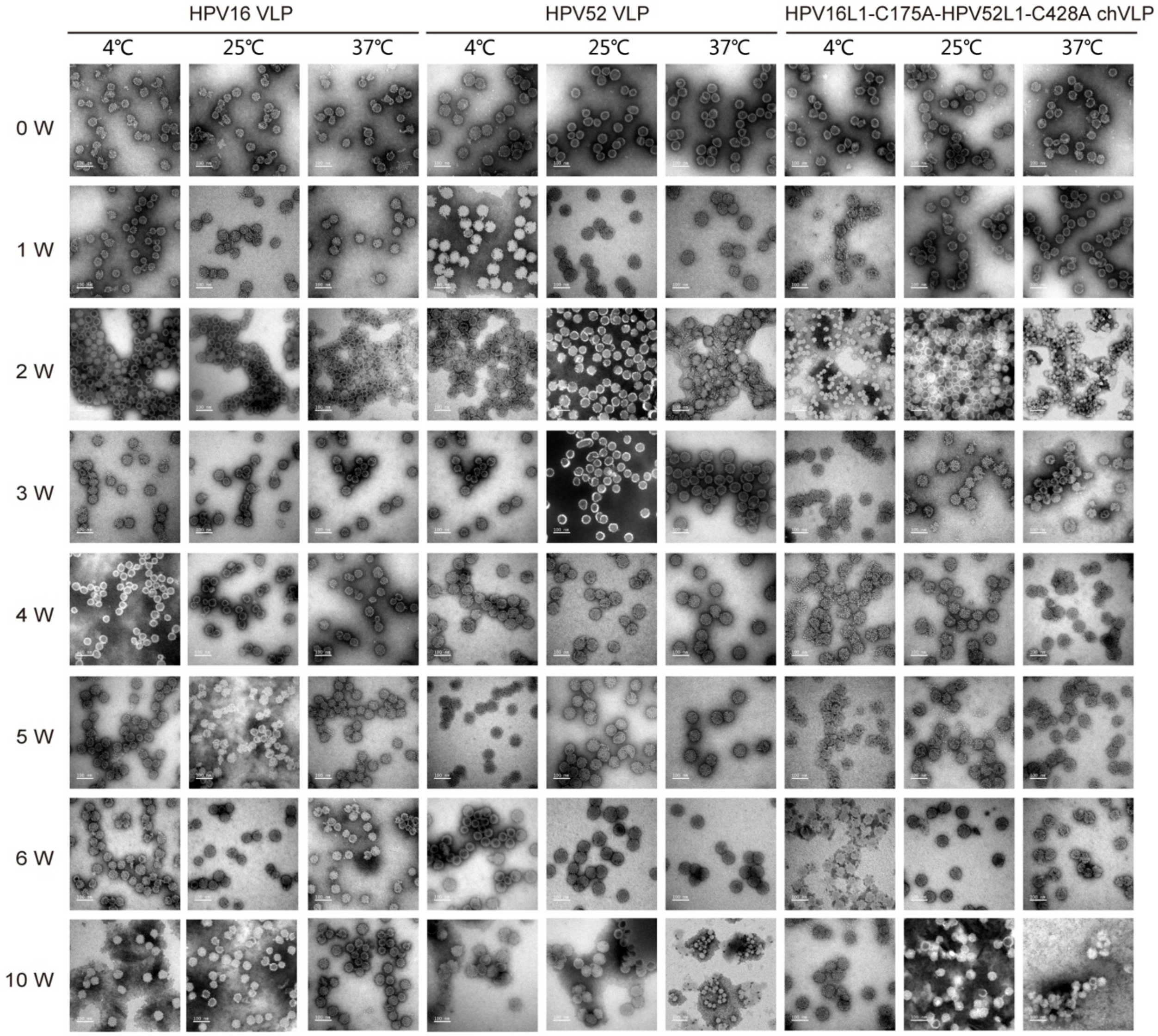

Supplementary Figure 9 Transmission electron microscopy (TEM) imaging to characterize the stability of HPV16, -52 VLPs and HPV16L1-C175A–HPV52L1-C428A chVLPs. All samples showed regular sphericity and were homogeneous in size, suggesting excellent stability for both chVLPs and WT VLPs. Scale bar, 100 nm. One representative image from three biological repeats is shown for each group.

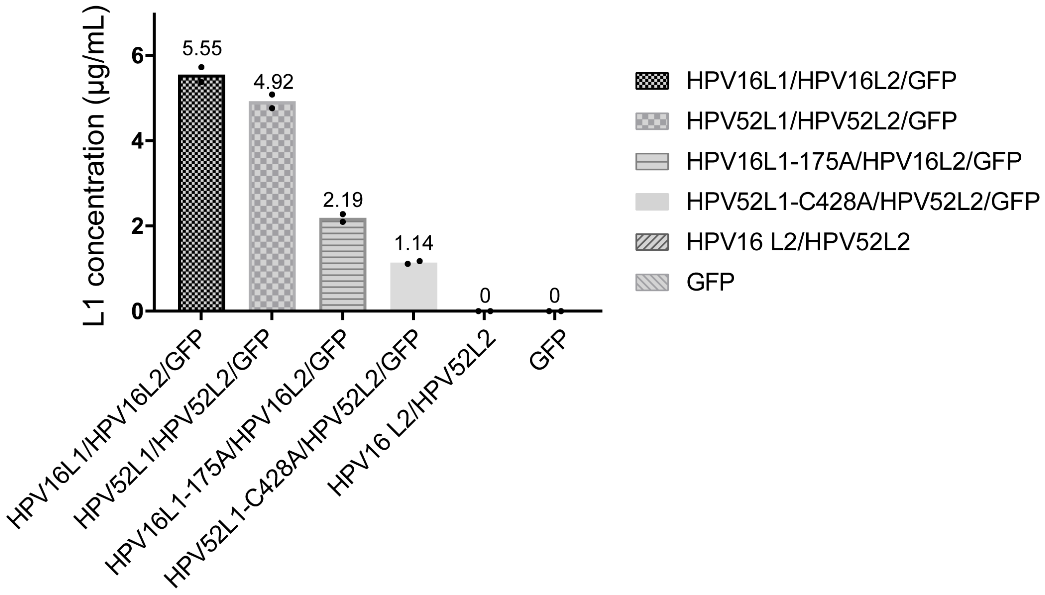

Supplementary Figure 10 The expression levels of HPV16L1, HPV52L1, HPV16L1-C175A and HPV52L1-C428A in 293FT cells were quantified using sandwich ELISA. HPV L2 and GFP served as the control. Data was presented as mean (n=2). Source data are provided as a source data file.

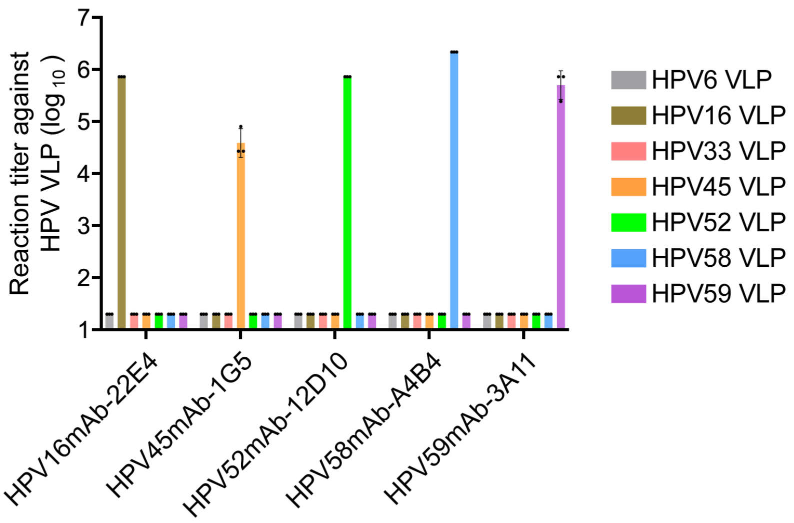

Supplementary Figure 11 Binding assay of HPV16, -45, -52, -58 and -59 genotype-specific mAbs to HPV L1 VLPs, determined by indirect ELISA and denoted as reactivity titer (*y*-axis). Bars and error bars indicate mean±SD (n = 3). Source data are provided as a source data file.

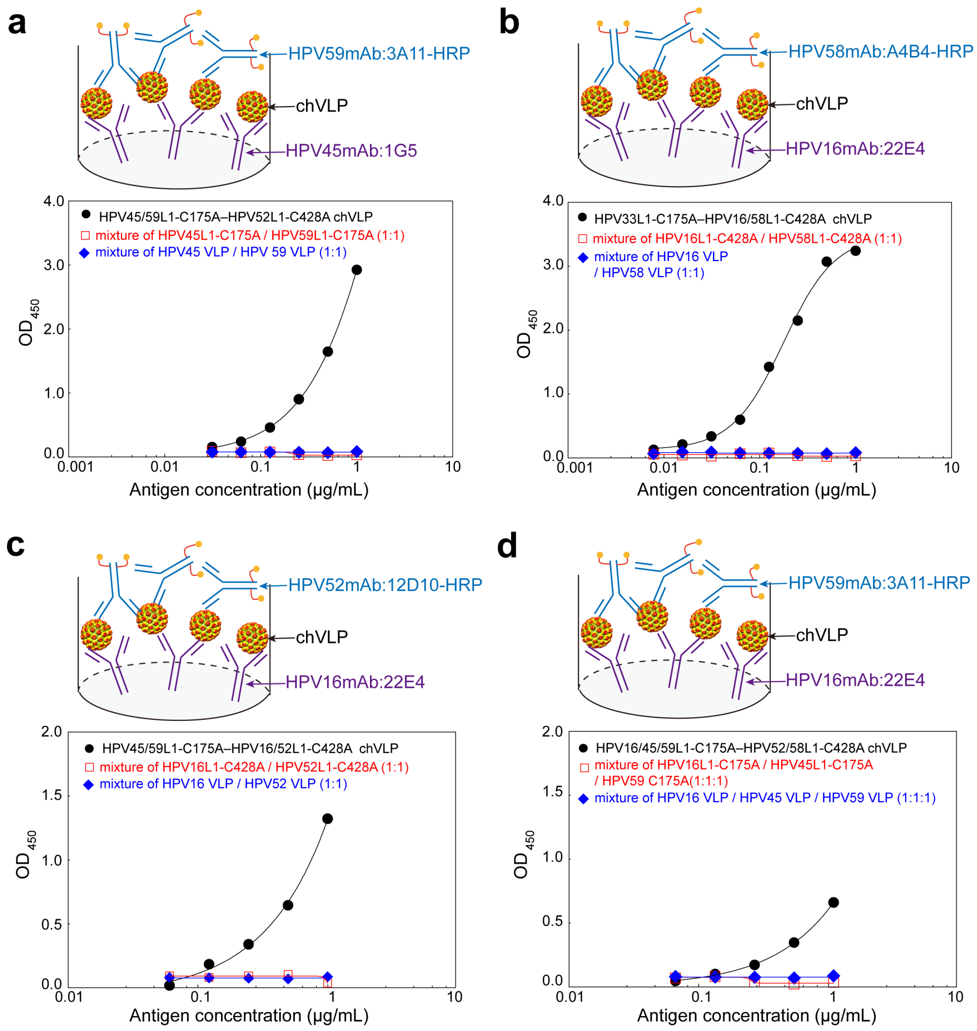

Supplementary Figure 12 Antigenicity of the chVLPs were determined using sandwich ELISA. (a) HPV45/59L1-C175A–HPV52L1-C428A chVLPs. 1G5 was used as the capturing antibody and 3A11-HRP as the detection antibody. (b) HPV33L1-C175A–HPV16/58-C428A chVLPs. 22E4 was used as the capturing antibody and A4B4-HRP as the detection antibody. (c) HPV45/59L1-C175A–HPV16/52L1-C428A chVLPs. 22E4 was used as the capturing antibody and 12D10-HRP as the detection antibody. (d) HPV16/45/59L1-C175A–HPV52/58-C428A chVLPs. 22E4 was used as the capturing antibody and 3A11-HRP as the detection antibody. Data were obtained from single experiment.

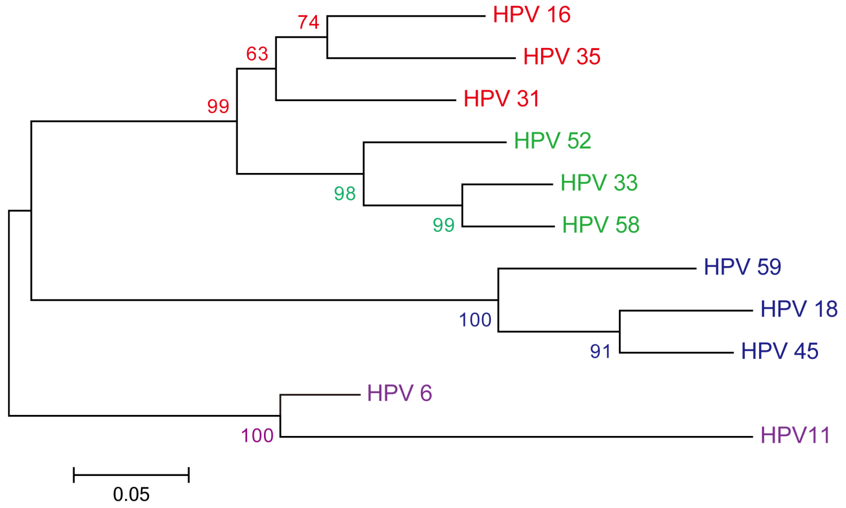

Supplementary Figure 13 Phylogenetic tree of HPV L1 proteins based on the amino acid sequences of 11 HPV types using the maximum likelihood method. The bar at the bottom provides a scale for the change in evolutionary lineages over time.

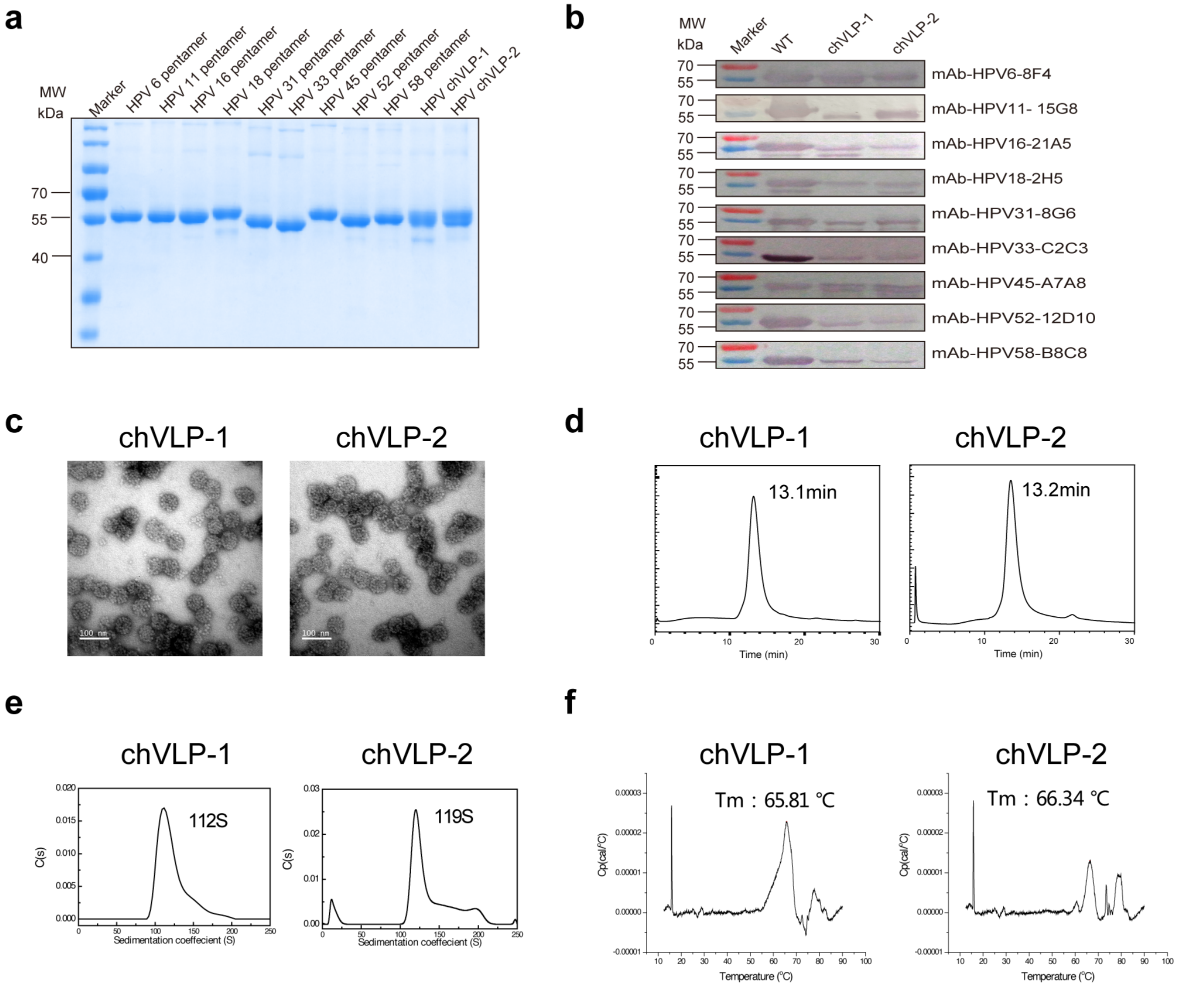

Supplementary Figure 14 Characterization of the nona-type chVLPs. Two forms of nona-type chVLPs were produced, referred to as chVLP-1 and chVLP-2, respectively: HPV16/18/31/45-C175A-HPV6/11/33/52/58-C428A and HPV6/11/33/52/58-C175A-HPV16/18/31/45-C428A chVLPs. (a, b) Each of the nine WT L1 pentamers and the two nona-type chVLPs were analyzed by reducing SDS-PAGE and western blotting using HPV-L1-specific mAbs (HPV6-8F4, HPV11-15G8, HPV16-21A5, HPV18-2H5, HPV31-8G6, HPV33-C2C3, HPV45-A7A8, HPV52-12D10 and HPV58-B8C8). (c) TEM analysis, Scale bar, 100 nm, (d) HPSEC, (e) AUC, and (f) DSC. For c, one representative image from three biological repeats is shown. The uncropped original scans can be found in Supplementary Fig. 18.

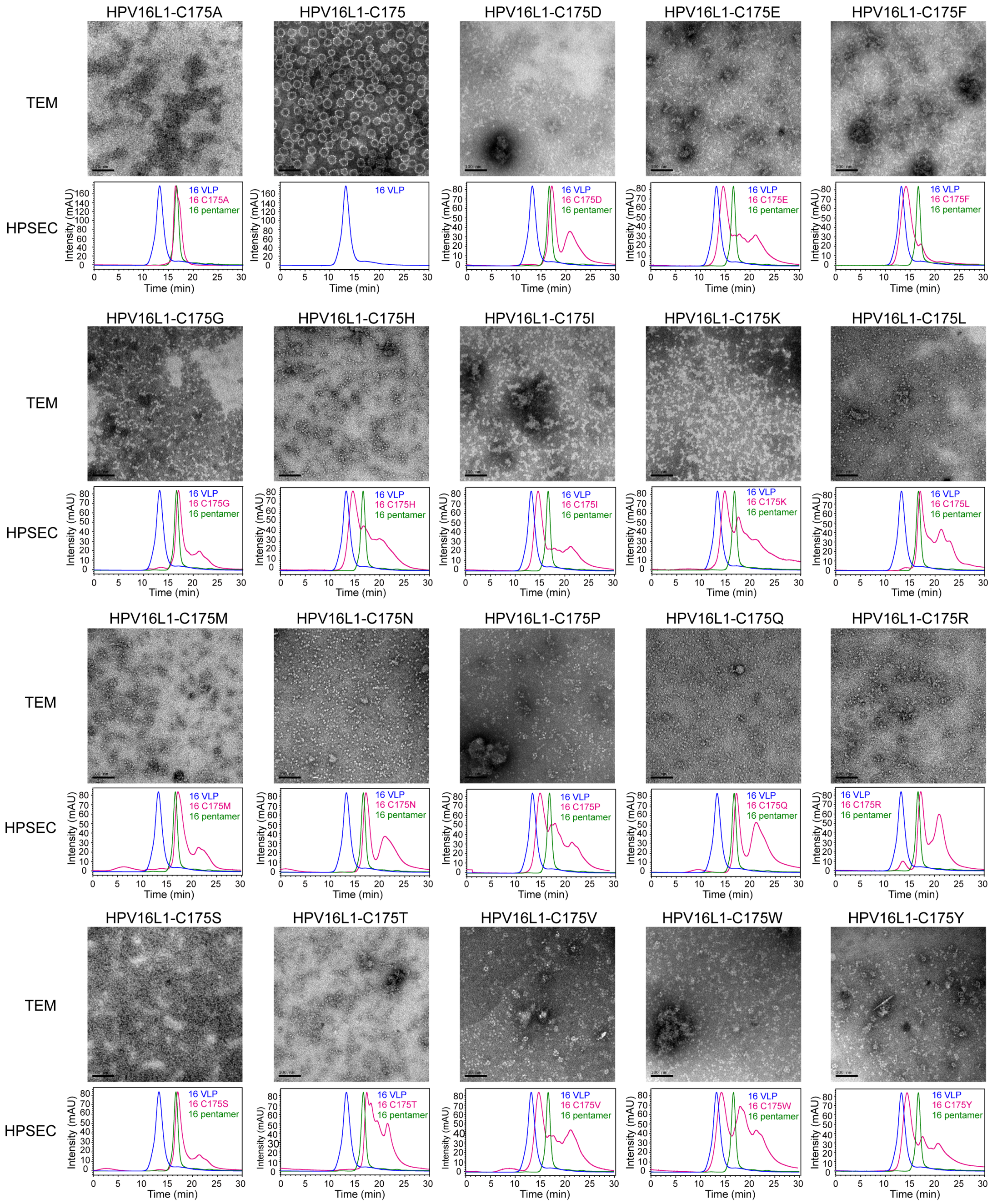

Supplementary Figure 15 Micrographs of negatively stained and high-performance size-exclusion chromatography (HPSEC) profiles of the saturated C175 mutants of HPV16 L1. Scale bar, 100 nm. One representative image from three biological repeats is shown.

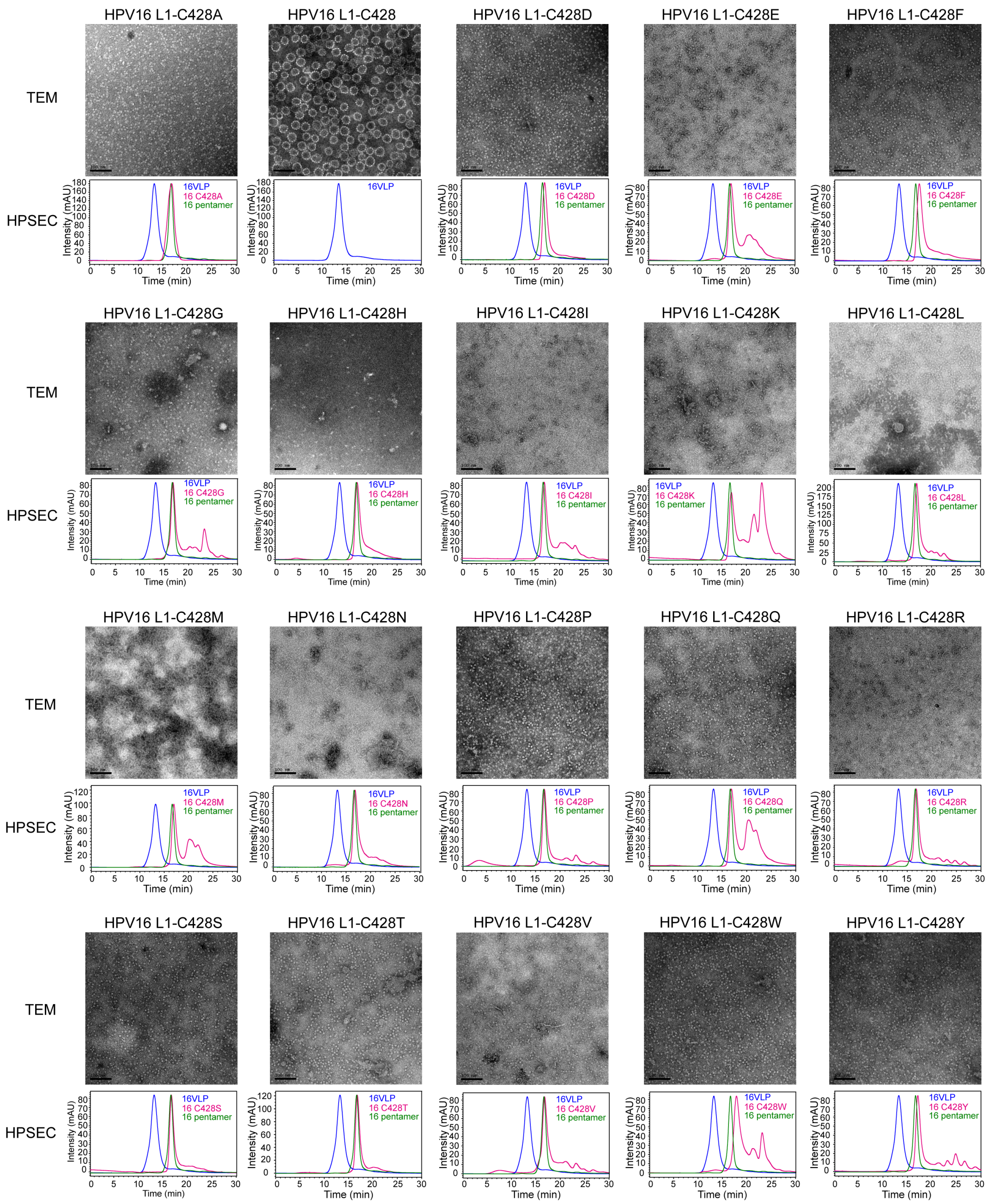

Supplementary Figure 16 Micrographs of negatively stained and high-performance size-exclusion chromatography (HPSEC) profiles of the saturated C428 mutants of HPV16 L1. Scale bar, 100 nm. One representative image from three biological repeats is shown.

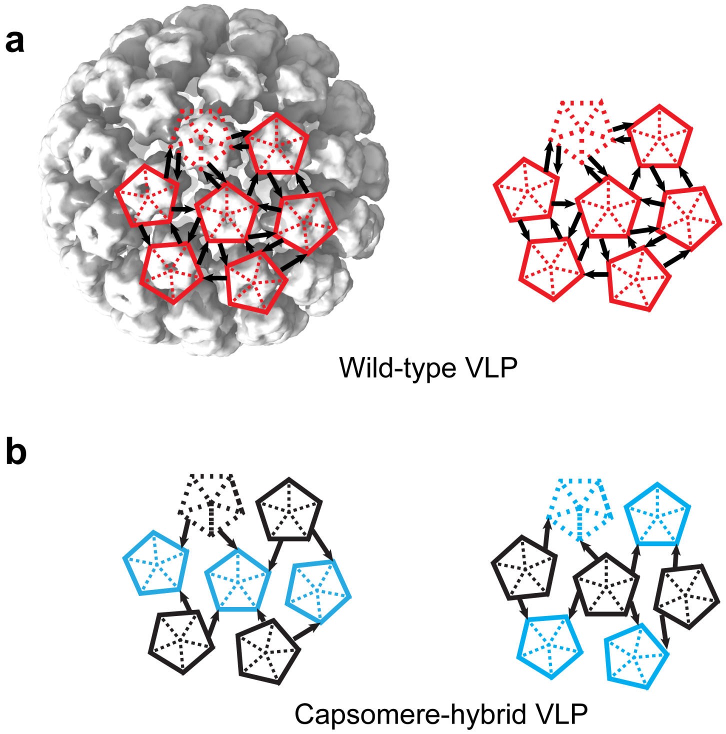

Supplementary Figure 17 Diagrams of the proposed pentamer packing in WT and capsomere-hybrid VLPs. (a) In the full-size capsids of HPV, 72 pentamers are arrayed at the vertices of a T=7d icosahedral lattice. Twelve pentamers (penton, dashed pentagon) are centered on a 5-fold symmetry axes, whereas the 60 other pentamers (only 6 are shown as open red pentagons) are at 6-coordinated positions. (b) For capsomere-hybrid VLPs, the two different types of mutated pentamers—HPV16 L1 C175A (black) and HPV52 L1 C428A (blue)—could be arrayed as two possible placements to maximize the number of disulfide bonds (black arrows). Compared with WT VLPs, only half of the disulfide bonds form.

Supplementary Figure 18 Uncropped Original Scans

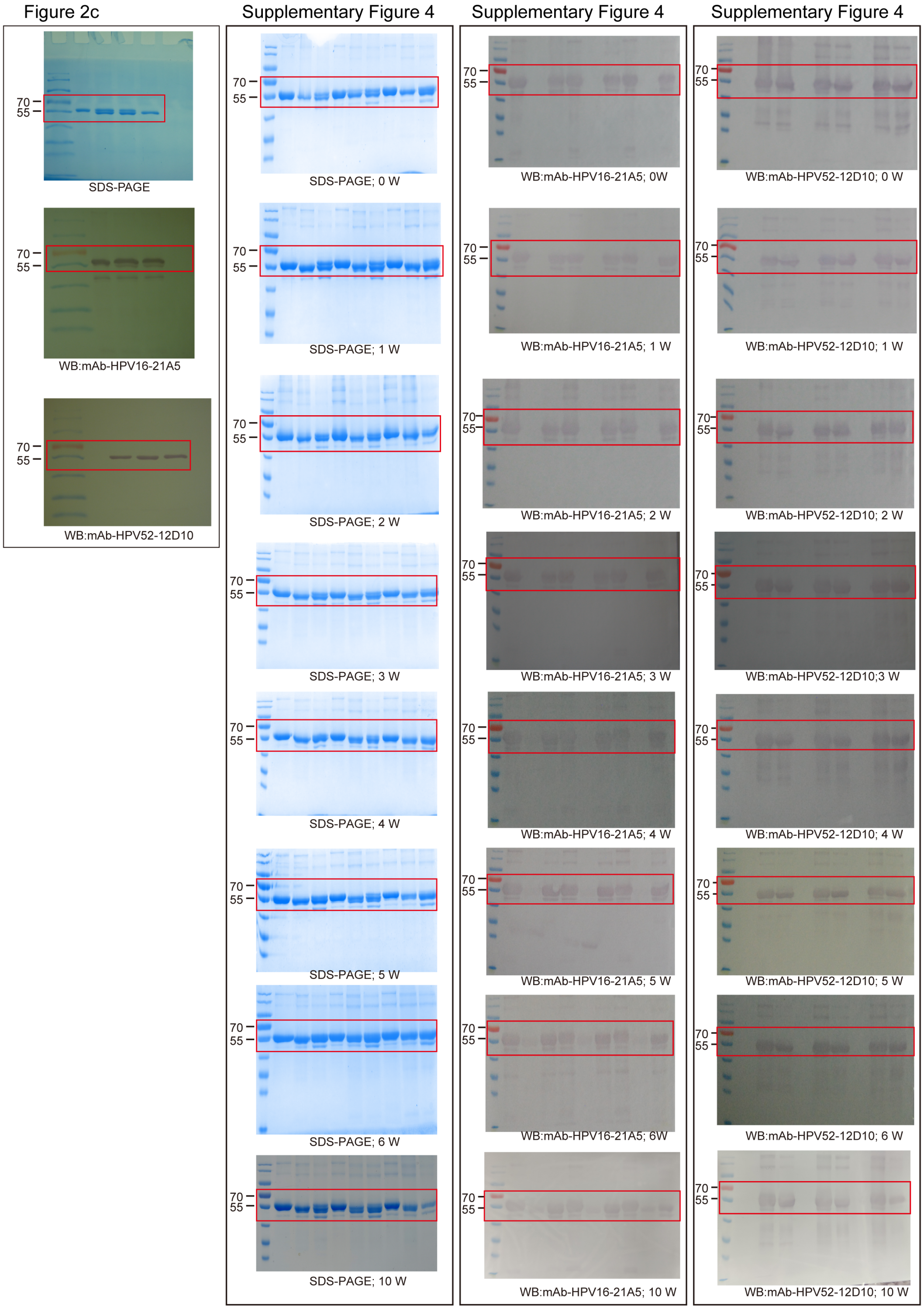

Uncropped Original Scans from Figure 2c and Supplementary Figure 4.

Supplementary Figure 14b

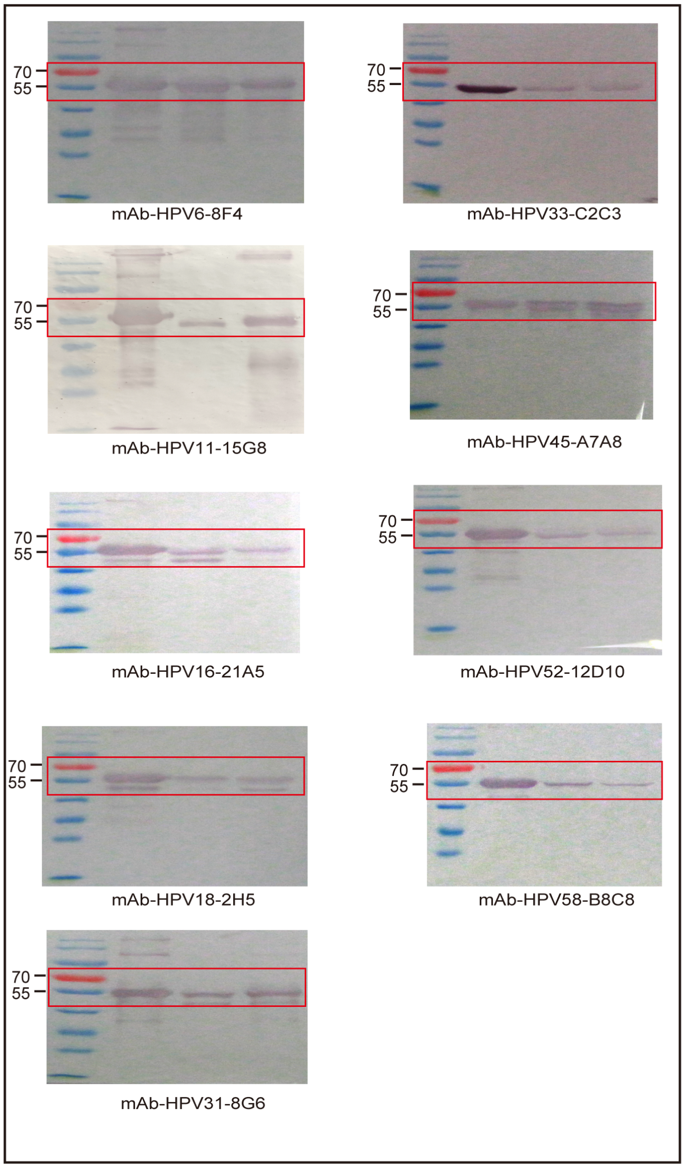

Uncropped Original Scans from Supplementary Figure 14b.

Supplementary Table 1 Cryo-EM Data Collection and Reconstructions.

	HPV16L1-C175A–HPV52L1-C428A chVLP (EMD-0878)
**Data collection and processing**	
Magnification	×93,000
Voltage (kV)	300
Electron exposure (e–/Å^2)	25
Defocus range (μm)	1.20-4.37
Pixel size (Å)	1.109
Symmetry imposed	I2
Initial particle images (no.)	325
Final particle images (no.)	278
Map resolution (Å)	26.1
FSC threshold	0.143
Map resolution range (Å)	25.0-30.0

Supplementary Table 2 Multi-type capsomere-hybrid VLPs used in Fig. 5 and Fig. 8

ch VLPs name	Multi-type Capsomere-hybrid VLPs formation
ch VLPs A	HPV45/59L1-C175A–HPV52L1-C428A
ch VLPs B	HPV16/33L1-C175A–HPV58L1-C428A
ch VLPs C	HPV16/59L1-C175A–HPV52L1-C428A
ch VLPs D	HPV 33L1-C175A–HPV 16/58L1-C428A
ch VLPs E	HPV 45L1-C175A–HPV 16/52L1-C428A
ch VLPs F	HPV 33/52L1-C175A–HPV 16/58L1-C428A
ch VLPs G	HPV 45/59L1-C175A–HPV 16/52L1-C428A
ch VLPs H	HPV 16/59L1-C175A–HPV 52/58L1-C428A
ch VLPs I	HPV 45/59L1-C175A–HPV 16/52/58L1-C428A
ch VLPs J	HPV 33/59L1-C175A–HPV 16/52/58L1-C428A
ch VLPs K	HPV 16/45/59L1-C175A–HPV 52/58L1-C428A
ch VLPs L	HPV 16/33/59L1-C175A–HPV 52/58L1-C428A
ch VLPs M	HPV 33/45/59L1-C175A–HPV 16/52/58L1-C428A
ch VLPs N	HPV 16/33/59L1-C175A–HPV 6/52/58L1-C428A
ch VLPs O	HPV 33/45/52/59L1-C175A–HPV 16/58L1-C428A
ch VLPs P	HPV 33/45/52/59L1-C175A–HPV 6/16/58L1-C428A
ch VLP-1	HPV16/18/31/45-C175A-HPV6/11/33/52/58-C428A
ch VLP-2	HPV6/11/33/52/58-C175A-HPV16/18/31/45-C428A

Supplementary Table 3 L1 composition and immunization dose of of the multi-type capsomere-hybrid VLPs

	Vaccine formulation (unit:µg)							
Group name	HPV6	HPV16	HPV33	HPV45	HPV52	HPV58	HPV59	Total dose
ch VLPs C	0	0.625	0	0	1.25	0	0.625	2.5
VLP mixture C	0	0.625	0	0	1.25	0	0.625	2.5
ch VLPs D	0	0.625	1.25	0	0	0.625	0	2.5
VLP mixture D	0	0.625	1.25	0	0	0.625	0	2.5
ch VLPs E	0	0.625	0	1.25	0.625	0	0	2.5
VLP mixture E	0	0.625	0	1.25	0.625	0	0	2.5
ch VLPs F	0	0.875	0.875	0	0.875	0.875	0	3.5
VLP mixture F	0	0.875	0.875	0	0.875	0.875	0	3.5
ch VLPs H	0	0.875	0	0	0.875	0.875	0.875	3.5
VLP mixture H	0	0.875	0	0	0.875	0.875	0.875	3.5
ch VLPs J	0	0.67	1.00	0	0.67	0.67	1.00	4.0
VLP mixture J	0	0.67	1.00	0	0.67	0.67	1.00	4.0
ch VLPs L	0	0.67	0.67	0	1.00	1.00	0.67	4.0
VLP mixture L	0	0.67	0.67	0	1.00	1.00	0.67	4.0
ch VLPs O	0	1.25	0.625	0.625	0.625	1.25	0.625	5.0
VLP mixture O	0	1.25	0.625	0.625	0.625	1.25	0.625	5.0
ch VLPs P	0.83	0.83	0.625	0.625	0.625	0.83	0.625	5.0
VLP mixture P	0.83	0.83	0.625	0.625	0.625	0.83	0.625	5.0

Supplementary Table 4 L1 composition and immunization dose of the nona-type chVLPs and control vaccines

Group name	Group number	Vaccine formulation (unit: µg)									
		HPV6	HPV11	HPV16	HPV18	HPV31	HPV33	HPV45	HPV52	HPV58	Dose
	1	1.5	2	3	2	1	1	1	1	1	13.5
**WT VLP-1**	2	0.15	0.2	0.3	0.2	0.1	0.1	0.1	0.1	0.1	1.35
	3	0.015	0.02	0.03	0.02	0.01	0.01	0.01	0.01	0.01	0.135
	4	1.5	2	3	2	1	1	1	1	1	13.5
**WT VLP-2**	5	0.15	0.2	0.3	0.2	0.1	0.1	0.1	0.1	0.1	1.35
	6	0.015	0.02	0.03	0.02	0.01	0.01	0.01	0.01	0.01	0.135
	7	1.5	2	3	2	1	1	1	1	1	13.5
**Gardasil 9**	8	0.15	0.2	0.3	0.2	0.1	0.1	0.1	0.1	0.1	1.35
	9	0.015	0.02	0.03	0.02	0.01	0.01	0.01	0.01	0.01	0.135
	10	0	0	6.75	6.75	0	0	0	0	0	13.5
**Cervarix**	11	0	0	0.675	0.675	0	0	0	0	0	1.35
	12	0	0	0.0675	0.0675	0	0	0	0	0	0.135
	13	1.5	2	3	2	1	1	1	1	1	13.5
**chVLP-1**	14	0.15	0.2	0.3	0.2	0.1	0.1	0.1	0.1	0.1	1.35
	15	0.015	0.02	0.03	0.02	0.01	0.01	0.01	0.01	0.01	0.135
	16	1.5	2	3	2	1	1	1	1	1	13.5
**chVLP-2**	17	0.15	0.2	0.3	0.2	0.1	0.1	0.1	0.1	0.1	1.35
	18	0.015	0.02	0.03	0.02	0.01	0.01	0.01	0.01	0.01	0.135
