# Supplementary material for: Rational design of a multi-valent human papillomavirus vaccine by capsomere-hybrid co-assembly of virus-like particles
Source: Nat Commun. 2020 Jun 5;11:2841. doi: 10.1038/s41467-020-16639-1 (PMC7275066; doi:10.1038/s41467-020-16639-1)
Supplement: Supplementary file 3 — Reporting Summary [file 41467_2020_16639_MOESM3_ESM.pdf]

nature research

Corresponding author(s): Shaowei Li

Last updated by author(s): May 4, 2020

Reporting Summary

Nature Research wishes to improve the reproducibility of the work that we publish. This form provides structure for consistency and transparency in reporting. For further information on Nature Research policies, see Authors & Referees and the Editorial Policy Checklist .

Statistics

For all statistical analyses, confirm that the following items are present in the figure legend, table legend, main text, or Methods section.

n/a	Confirmed	
☐	☒	The exact sample size (n) for each experimental group/condition, given as a discrete number and unit of measurement
☐	☒	A statement on whether measurements were taken from distinct samples or whether the same sample was measured repeatedly
☐	☒	The statistical test(s) used AND whether they are one- or two-sided<br>*Only common tests should be described solely by name; describe more complex techniques in the Methods section.*
☒	☐	A description of all covariates tested
☒	☐	A description of any assumptions or corrections, such as tests of normality and adjustment for multiple comparisons
☐	☒	A full description of the statistical parameters including central tendency (e.g. means) or other basic estimates (e.g. regression coefficient) AND variation (e.g. standard deviation) or associated estimates of uncertainty (e.g. confidence intervals)
☐	☒	For null hypothesis testing, the test statistic (e.g. F, t, r) with confidence intervals, effect sizes, degrees of freedom and P value noted<br>*Give P values as exact values whenever suitable.*
☒	☐	For Bayesian analysis, information on the choice of priors and Markov chain Monte Carlo settings
☒	☐	For hierarchical and complex designs, identification of the appropriate level for tests and full reporting of outcomes
☒	☐	Estimates of effect sizes (e.g. Cohen's d, Pearson's r), indicating how they were calculated

Our web collection on statistics for biologists contains articles on many of the points above.

Software and code

Policy information about availability of computer code

Data collection	The commercial software EPU (Themor Fisher, https://www.fei.com/software/epu/) was used for cryoEM data collection.
Data analysis	All software used for data analysis in this study were available online:<br>1. Prism 7.0 (https://www.graphpad.com/scientific-software/prism/): graphing, comprehensive curve fitting, statistics, and data organization;<br>2. MotionCor2 (http://msg.ucsf.edu/em/software/motioncor2.html): micrography collection;<br>3. Gctf (https://en.wikibooks.org/w/index.php?title=Software_Tools_For_Molecular_Microscopy&stable=0#Gctf): ctf estimation;<br>4. EMAN2 (http://blake.bcm.tmc.edu/EMAN2/): particle picking<br>5. Relion2.0 (http://www2.mrc-lmb.cam.ac.uk/relion): CryoEM map reconstruction;<br>6. AUTO3DEM (http://cryoem.ucsd.edu/wikis/software/start.php?id=auto3dem:home):CryoEM initial model create and 3D reconstruction.<br>7. Chimera (http://www.cgl.ucsf.edu/chimera): Density maps or structural models based visualization, segmentation and movies generation;<br>8. MEGA 10.1.7 (https://www.megasoftware.net): the phylogenetic analysis of HPV L1 aa sequences

For manuscripts utilizing custom algorithms or software that are central to the research but not yet described in published literature, software must be made available to editors/reviewers. We strongly encourage code deposition in a community repository (e.g. GitHub). See the Nature Research guidelines for submitting code & software for further information.

Data

Policy information about availability of data

All manuscripts must include a data availability statement. This statement should provide the following information, where applicable:

- Accession codes, unique identifiers, or web links for publicly available datasets
- A list of figures that have associated raw data
- A description of any restrictions on data availability

All data supporting the findings of this study are available from the corresponding author on reasonable request. The EM density map for HPV16L1-C175A–HPV52L1-C428 chVLP has been deposited in the Electron Microscopy Data Bank (accession code EMD-0878, https://www.ebi.ac.uk/pdbe/entry/emdb/EMD-0878). The source data underlying Figs. 6, 7, 8 and Supplementary Figs. 3c, 10 ,11 are provided as a Source Data file.

Field-specific reporting

Please select the one below that is the best fit for your research. If you are not sure, read the appropriate sections before making your selection.

☒ Life sciences ☐ Behavioural & social sciences ☐ Ecological, evolutionary & environmental sciences

For a reference copy of the document with all sections, see nature.com/documents/nr-reporting-summary-flat.pdf

Life sciences study design

All studies must disclose on these points even when the disclosure is negative.

Sample size	Sample size for each experiment is indicated in the figure legend for each experiment. The sample size was chosen based on previous experience for each experiment . No statistical methods were used to predetermine sample size.
Data exclusions	No data were excluded from the analysis.
Replication	All experimental findings were reliably reproducible.
Randomization	Animals were randomly divided into experimental groups.
Blinding	Investigators were not blinded to groups during experiments. Data reported for mouse experiments are not subjective but rather based on quantitative immunogen and neutralizing antibody titers.

Reporting for specific materials, systems and methods

We require information from authors about some types of materials, experimental systems and methods used in many studies. Here, indicate whether each material, system or method listed is relevant to your study. If you are not sure if a list item applies to your research, read the appropriate section before selecting a response.

Materials & experimental systems

n/a	Involved in the study	
☐	☒	Antibodies
☐	☒	Eukaryotic cell lines
☒	☐	Palaeontology
☐	☒	Animals and other organisms
☒	☐	Human research participants
☒	☐	Clinical data

Methods

n/a	Involved in the study	
☒	☐	ChIP-seq
☒	☐	Flow cytometry
☒	☐	MRI-based neuroimaging

Antibodies

Antibodies used

Antibodies purchased from commercial suppliers:
1: Goat anti-mouse alkaline phosphatase-conjugated antibodies, Abcam, #ab97020, 1:5000 dilution.
2: HRP-conjugated goat antimouse IgG antibody, Abcam, #ab19195, 1:5000 dilution.

Antibodies generated in our laboratory:
1: Anti-HPV16 antibodies: PA1, PD4, PB2, 8A9, PD1, 1D12, 19E9, 21A5, PD3, V5, 17A9, PD5.
2: Anti-HPV52 antibodies: 17C9, 6H12, 16D5, 10H2, 6, 29, 19D7, 9F12, 11F1, 4B3, 9F6, 12D10.
3: Anti-chVLPs antibodies: 2F4, 7F6, 15B10, 5A2, 7G5, 3A6, 4D3, 10C3.
4: Anti-HPV33 antibody: 12C8.
5: Anti-HPV45 antibody: 3F11.

	6: Anti-HPV58 antibody: A4B4, 7F2, A1H6.<br>7: Anti-HPV59 antibody: 13A6.<br>All these antibodies were diluted with the start concentration of 1 μg/mL.
Validation	The activities of antibodies were confirmed by indirect HPV VLP-based ELISA and pseudovirus-based neutralization assays before use.

Eukaryotic cell lines

Policy information about cell lines

Cell line source(s)	293FT cell line was purchased from Thermo Fisher (Catalogue number: R70007).
Authentication	None of the cell lines were authenticated.
Mycoplasma contamination	All cell lines tested negative for mycoplasma contamination.
Commonly misidentified lines<br>(See ICLAC register)	No commonly misidentified line was used.

Animals and other organisms

Policy information about studies involving animals; ARRIVE guidelines recommended for reporting animal research

Laboratory animals	The 6-8 weeks age specific pathogen-free (SPF) BALB/c female mice were used in this study. The housing conditions are described in the methods section of the manuscript.
Wild animals	The study did not use wild animals.
Field-collected samples	No field-collected sample was used in this study.
Ethics oversight	All animal experimental protocols were reviewed and approved by the Animal Care and Use Committee of Xiamen University. The manipulation and vaccination of animals strictly adhered to and complied with the guidelines provided by XMULAC.

Note that full information on the approval of the study protocol must also be provided in the manuscript.
